# A rapid and intelligent designing technique for patient-specific and 3D-printed orthopedic cast

**DOI:** 10.1186/s41205-016-0007-7

**Published:** 2016-12-01

**Authors:** Hui Lin, Lin Shi, Defeng Wang

**Affiliations:** 1 Research Center for Medical Image Computing, Department of Imaging and Interventional Radiology, The Chinese University of Hong Kong, Shatin, NT Hong Kong; 2 Department of Medicine and Therapeutics, The Chinese University of Hong Kong, Shatin, NT Hong Kong; 3 Chow Yuk Ho Center of Innovative Technology for Medicine, The Chinese University of Hong Kong, Shatin, NT Hong Kong; 4 Shenzhen Research Institute, The Chinese University of Hong Kong, Shenzhen, China

**Keywords:** Orthopedic cast, 3D-printed, Rapid, Intelligent, Patient-specific, Hygienic, Ventilated

## Abstract

**Background:**

Two point four out of 100 people suffer from one or more fractures in the course of average lifetimes. Traditional casts are featured as cumbersome structures that result in high risk of cutaneous complications. Clinical demands for developing a hygienic cast have gotten more and more attention. 3D printing technique is rapidly growing in the fabrication of custom-made rehabilitation tools. The objective of this study is to develop a rapid and intelligent modeling technique for developing patient-specific and hygienic orthopedic casts produced by 3D printing technologies.

**Results:**

A cast model is firstly created from a patient’s image to develop patient-specific features. A unique technique to creating geometric reference has been developed to perform detail modeling cast. The cast is modeled as funnel-shaped geometry to create smooth edges to prevent bruises from mild movements of injured limbs. Surface pattern includes ventilation structure and opening gap for hygienic purpose and wearing comfort. The cast can be adjusted to accommodate swelling from injured limbs during treatment. Finite element analysis (FEA) is employed to validate the mechanical performance of the cast structure and identify potential risk of the structural collapse due to concentrated stresses. The cast is fabricated by 3D printing technology using approval material.

**Conclusions:**

The 3D-printed prototype is featured as super lightweight with 1/10 of weight in compared with traditional alternatives. Medical technicians with few experiences can design cast within 20 min using the proposed technique. The image-based design minimizes the distortion during healing process because of the best fit geometry. The highly ventilated structure develops hygienic benefits on reducing the risk of cutaneous complications and potentially improve treatment efficacy and increase patients’ satisfactions.

## Background

Bone fracture occurs in general population resulting from mechanical impact or bone diseases. Plaster or fiberglass cast have been employing for the treatment of most fracture patients. Traditional orthopedic casts or orthoses are produced by body-based contacting model [1]. The bottom mold for a cast is generated from surface shapes of injury limbs and filled up with plaster. Thermoplastic material, PE (Polyethylene) and CPP (copolymer polypropylene), are laid on a mold and removed after cooling down for the formation of an orthopedic cast [2–4]. Fracture patients wear plaster splints after surgery followed by a cast for further recovery. Those casts develop several skin diseases and potential bone and joint injuries due to heavy structure and poor ventilation. Moreover, patients suffer mechanical pressures during the mold manufacture and multi-reproduction of physical molds are unfeasible [3].

3D printing technology is a rapid growing manufacture technique for producing a complex physical model in term of 3D a digitizing model [5–10]. It recently has been extensively applying on surgical practices and medical training [8, 11–15]. The rapid manufacture of the physical model from medical images provides technical means with minimal invasion for medical planning and treatment [7, 8, 16–20]. Custom-made rehabilitation tools produced from 3D printing technique have been reported in the new development of orthopedic cast [8, 21]. Conventional custom-fit casts generate the surface geometry from subject’s injured limbs. Mavroidis et al. developed a novel engineering process using rapid prototyping technique for creating patient-specific ankle-foot orthosis [4]. The process basically included, 3D scanning injury ankle and leg, designing in a Computer Aided Design (CAD) software and exporting STereoLithography (STL) file, and manufacture using a 3D-printing technique [3, 4]. The engineering method is also employed in the design and manufacture for wrist orthosis [3, 21]. The upper limb was scanned and 3D digitized. The polygonal data, STL file, was generated and input into a CAD software for designing a desired model. The designing model was output for rapid prototyping.

Some novel concepts for potential substitutes for plaster cast and manufactured by 3D printing technology are reported [1, 3, 4]. Jake Evill proposed a new design for orthoses named as Cortex, a custom-fitted web structure [3]. The mesh-like structure forms an artistic surface pattern with treatment consideration by changing the webby density, more solid at the region of fracture. Another idea proposed by Deniz Karasahin developed a similar model as Cortex but mounted an ultrasound device for promoting the therapeutic process. Those new designs are fabricated by 3D printing technique using environment-friendly material [2, 4]. Cast geometries are generated from 3D scan provide patient-specific models that offer wearing comfort and fashionable appearance. The mesh-like structure definitely presents excellent ventilation. However, the weak strength of the structure is present for supporting the injury limbs. Mechanical impact with low intensity may easily break the webby beam. In addition, the webby shape is most likely to develop crack and fatigue failure due to the slender connecting bar. Those fancy designs are still in technical assessment without any clinical application and approval.

A hybrid model for custom-fit wrist orthosis that combined the webby frame with shell cover to enhance the structural strength as well as keep ventilation [2, 3]. The design process basically included modeling inner frame and outer cover via a CAD system. This new model improved the stiffness and prevented the structure from breaking. However, an experienced CAD engineer was involved in creating appropriate engineering structure. This new design is a concept model without any clinical application.

Despite the technical advantage and economic potential, 3D-printing technologies have not become the primary mean in the fabrication of orthopedic cast [2]. The significant technical expertise is required for designing a cast and high cost and time for both design and fabrication are present. In order to perform CAD process, the scanned data of subject’s limbs must be converted into specific CAD file with modification of the geometry. An experienced CAD engineer is required for creating the model and converting the model CAD file to STL file for 3D-printing [3, 4]. The entire design process is manual and time cost. As reported in the literature, it took over three hours to design a wrist orthosis [3].

Clinical demands for developing a cast with good ventilation, light weight, and automatic design process and few requirements of expertise, have been getting more and more attention. The medical application of 3D printing is increasing for its rapid manufacture and cost-effective benefits. The growing technologies on 3D printing make it possible on the fabrication of complex geometric model presenting in orthopedic casts and significantly reduce the manufacturing time and cost [8, 14]. The objective of this study is to develop a rapid designing technique for creating patient-specific and hygienic orthopedic cast. 3D-printed technologies are used in the fabrication of the proposed design.

## Methods

The rapid modeling technique includes several algorithms for automatically generating geometric features. The proposed rapid modeling technique is a step-by-step procedure and basically include following steps:

- Develops basic model from image-based patient data
- Computes geometric reference of cast data
- Models flare edges
- Builds cast surface pattern
- Creates solid model of the cast

### Modeling data and computing geometric reference

**Fig. 1:**
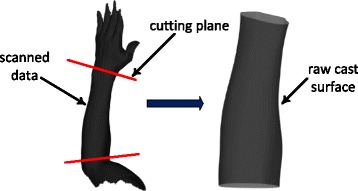
The scanned data of a limb and a raw cast surface cut from the data

We utilize the photometric scanner, Artec Eva and Artec Space Spider (Luxembourg), to scan the injured limb. Patients should be placed in an appropriate position for obtaining the adequate data in terms of requirements of image reconstruction. The surface geometry of limb is digitized and transferred as polygonal STL file with over 200,000 points and 400,000 triangle elements (Fig. 1). The number of point and element vary widely from one anatomic site to another. The initial cast surface model is generated from clipping the raw body data as shown in Fig. 1. The clipping location is determined by orthopedic technicians in accordance with the injury site. It should be noticed that clipping plane may not be visually perpendicular to the cast surface. In order to create a fine cast model with visually perpendicular end plane, a computation of centerline is proposed.

**Fig. 2:**
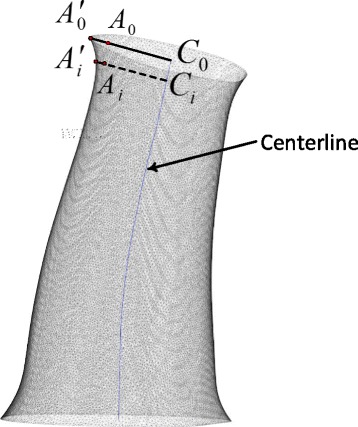
Generation of flare opening. Extending coefficients are applied on the surface points near the opening end and funnel-shaped opening end are developed

Creating geometric reference from the raw data is one of the most important steps to further build cast model. The geometric reference is a curve running through the central area and termed as the centerline of the data (Fig. 2). The computation of centerline and clipping fine edge are described as follows.

i) Centerline is geometrically defined as the shortest path to connect two endpoints of a tube-like cast. There are many mathematical definitions of centerline which is an intuitive central path. It should be noted that the centerline is bounded on the Voronoi diagram of the cast and is composed of points centered the maximal inscribed sphere. A huge amount of literature described the Voronoi diagram [22, 23]. In this study, the centerline is calculated by The Vascular Modeling Toolkit (VMTK, Orobix Srl).
ii) Once the centerline is created, it is used to define reference planes to perform fine clipping. The centerline is a spline line and the tangent vectors vary along the spline line. A clipping plane is defined by a normal vector, the average of a series of tangent vectors from an endpoint of the centerline. In this study, we used tangent vectors of 5 successive points started from an endpoint to calculate the normal vector. Two new clipping planes are generated in this step.
iii) Two new clipping planes are applied to the unclipped raw body surface, e.g. body data acquired from 3D scanner, to perform a re-clipping process. The two clipping planes locate at two endpoints of the centerline and a fine cast surface model is obtained.

### Generation of flare edges

Flare opening for a tube-like cast geometry creates a funnel-shaped end. Flare edges are required by orthopaedists for the consideration of wearing comfort and safety and modeled in both end sides of the fine cast model. The funnel-shaped geometry with round corner produces a smooth touching surface preventing injuries from the usual movement of part of injured limbs such as the wrist. Points on the surface areas near the opening edges are stretched by applying extending vectors. The mathematical form can be described as:

1 $$ {\overset{\rightharpoonup }{A^{\prime}}}_i-{\overset{\rightharpoonup }{C}}_i={b}_i\left({\overset{\rightharpoonup }{A}}_i-{\overset{\rightharpoonup }{C}}_i\right) $$

Where *C*_*i*_(*i* = 0, 1, … *n*) are central points on the centerline. *C*_*i*_ is the point nearest the original surface vertex point *A*_*i*_. ${\overset{\rightharpoonup }{A^{\prime}}}_i$ is the new vertex point stretched from *A*_*i*_. *b*_*i*_ is the extending coefficient with respect to points same layer as *A*_*i*_ (Fig. 2). Values of coefficient *b*_*i*_ linearly vary along the centerline. The maximum extending coefficients are applied on the opening ends of the tube-like cast. Not all surface points are applied the extending factor. In this project, surface points with distances 3 ~ 5 mm to the clipping plane are applied extending coefficient. There is no exact standard to determine the values of coefficients. The maximal coefficient is 1.15 using in this project. Funnel-shaped opening ends are more or less visual and different values of the maximal coefficient with orthopedic feasibility are acceptable. Flare shapes are generated on both sides of the cast in this step.

### Cast surface pattern

**Fig. 3:**
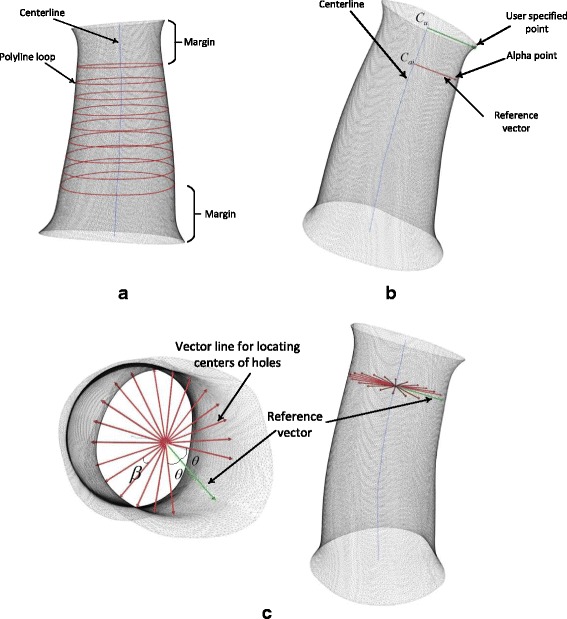
**a** Centerline is sliced as segments with equal distance and generated as a number of polyline loops on the cross section of the cast surface. **b** User-specified point is selected by a technician for locating the opening gap. Alpha point is a reference point for further locating centers of holes. **c** A bundle of vector lines are created for locating the hole centers

The following step is to create surface pattern including the ventilation structure and opening gap for mechanical assembly and adjustment purpose. The ventilation holes are uniformly distributed on the free surface of the cast. An algorithm is developed for performing this step automatically. The algorithm firstly averages the tangent vectors along the centerline, which is a spline line. It then uses the average of the tangents as the normal vector of the cutting planes. The total length of the centerline is computed by integration of all micro-segments that generate the centerline. The effective length of the centerline is defined by subtracting two marginal lengths from the total length as illustrated in Fig. 3a. The marginal lengths on both opening ends of the cast are specified by medical engineers in terms of the corresponding standard or experiences. The effective centerline, which removes two marginal segments from the centerline, is divided by equal distance segments and points in-between segments are extracted. Cutting planes are created at each points in-between segments and applied on slicing the casting surface to give polyline loops (Fig. 3a).

Ventilation holes are distributed on the free surface of cast uniformly in case the stress concentration and weak structure. However, a blank area on the cast surface should be reserved for creating an opening gap running through the cast surface for the consideration of the further mechanical assembly design. There are no holes drilling in this area. An orthopedic engineer selects a point call user-specified point as a marker for creating a blank area (Fig. 3b). The mapping point on the centerline *C*_*u*_ with the closest distance to the user specified point is determined and a vector line starting from *C*_*u*_ to user specified point is created. *C*_*ai*_(*i* = 1, 2 …, *n*) is a point on the centerline and locating at a cutting plane for generating corresponding polyline loop (Fig. 3b). An alpha point is defined as the references point locating at a polyline loop and served as the starting point to position the centers of holes along this loop. To determine the alpha point, a vector line is created to pass through *C*_*ai*_ and parallel with the vector line passing through *C*_*u*_. The alpha point is selected from the polyline loop with the closest distance to the vector line. Each cutting plane or each polyline loop has a vector line to be a reference vector as shown in Fig. 3b.

Each reference vector is used to create a bundle of vector lines for further locating the centers of holes. In terms of technical needs, the number of holes for each slice is pre-defined. In order to reserve a blank area as mentioned above, an angle formed by two vectors, i.e. reference vector and start vector line, is defined. The value of the angle is *θ* (Fig. 3c). In addition, the symmetric end vector line corresponding to the reference vector is also defined with the same angle *θ* between those two vectors. A circular pattern is applied to create a bundle of vector lines from the start vector line to the end vector line. The number of vector lines is equal to the required number of holes for each slice. Equal angle between two adjacent vector lines is required (Fig. 3c) and calculated as follows:

2 $$ \beta =\frac{\left(360-2\cdot \theta \right)}{m-1} $$

where *β* is the angle between two adjacent vectors and *m* is the number of holes for each slice. The centers of holes are located at the polyline loop with the closest distance to the vector lines. Each vector line determines one hole center (Fig. 3c).

**Fig. 4:**
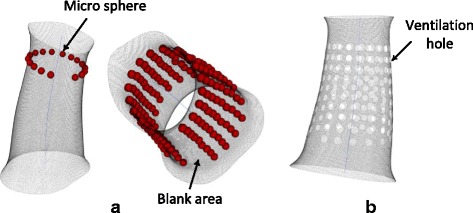
**a** A micro sphere is created in each center for modeling a hole. **b** The ventilation holes distribute on the free cast surface

Once centers of holes are selected, a number of spheres with those centers are modeled as shown in Fig. 4a. Those spheres have the same diameter, which value is determined based on technical needs. 2 or 3 mm of the diameter is suggested for upper or lower limbs. Values may vary on special cases depending on clinical application. Ventilation holes are created via geometrically Boolean operation between three-dimensional spheres and the free surface of the cast. This operation subtracts elements inside the sphere from the cast surface. It then develops a cast surface with uniformly distributed ventilation holes as displayed in Fig. 4b. The bigger size of the holes, the lighter weight of the structure will be built. However, the big size of the hole suggests that a relatively weak structure is developed due to less solid areas keeping in the structure. Engineering assessment of strength such as finite element analysis is recommended for validation.

An opening gap is for assembling and disassembling cast and makes the cast adjustable during the treatment of an injured limb. The path of the opening gap is not a straight line or an intersection line given by a flat plane and the cast surface. Geometric varieties of cast surface make it difficult to simply create the path via extracting intersection line between a flat plane and cast surface. A geodesics-based approach is employed in this study to model a curve as the path of the gap.

**Fig. 5:**
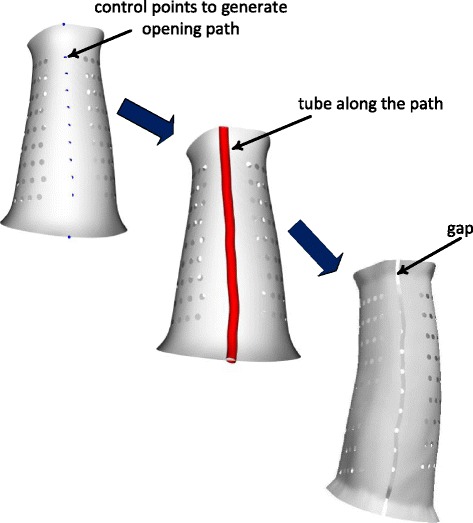
A control point set is created for generating the path of opening gap. A tube is modeled along the path and for further creating opening gap via Boolean operation

The previous steps created a marker specified by a user on the cast surface. Based on the marker point, an array of alpha points is selected in previous steps as illustrated in Fig. 3b. Those points are added to the control points list. It should be noted that more control points should be added to the list to ensure that those control points can run through cast surface along the longitudinal direction. The available control points have two end points that are employed as two reference points to locate the two end points on two opening flare edge. Those two end points have the closest distances to two reference points respectively. After obtaining two end points, those points are added to the control point set to generate a new point set, which will be used to create the profile of an opening gap (Fig. 5). Dijkstra geodesics are calculated based on the control points [24, 25]. Those geodesics create an initial path of the opening gap. The initial path will be a zigzag-like line that will not have engineering rationality due to high risk of developing crack and fatigue and poor wearing comfort.

The next step is to build a smooth curve as the path for the opening gap. Control point set will be added more points where those points are located at geodesics. The new set multiplies the points for modeling an accurate and rational path line. A spline line passing through all points in the new set is built and run through the cast surface. The spline line is defined as the path of the opening gap.

The circle is created at one end point of the spline line and sweep along the path to generate a tube Fig. 5. The spline line is then served as the centerline of the tube. A small size of the tube would develop a small gap which would better enclose injury limbs. The smaller size of the tube, the better cast structure is modeled. For the consideration of the manufacturing feasibility, i.e. accuracy of 3D printing, the diameter is set as around 2 mm. A parametric tube is created for adjustments of the gap space. Once a tube is built, the opening gap is developed by performing Boolean subtraction between the tube and cast surface as displayed in Fig. 5.

### Cast solid model

**Fig. 6:**
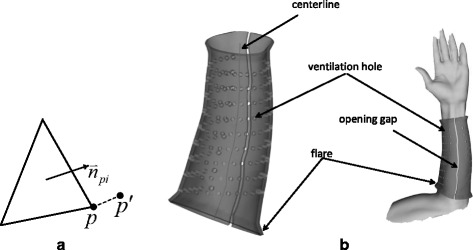
**a** Offset a surface vertex along a normal vector of a patch. **b** The modeling technique of the cast including centerline, ventilation hole, opening gap, and flare. Thickness of the cast is modelled in the last step

The thickness of the cast is built by offsetting the cast surface. The cast surface is composed of a large number of triangle elements. Each piece of elements has its normal vector calculated from the cross product of vectors along any two sides of the element. The normal vector pointing to outside cast surface is considered as the reference direction for offsetting (Fig. 6a). The offsetting process can be mathematically written as:

3 $$ \overset{\rightharpoonup }{p^{\prime }}=\overset{\rightharpoonup }{p}+t\cdot {\overset{\rightharpoonup }{n}}_{pi} $$

where $\overset{\rightharpoonup }{p}$ is an original vertex of an element, $\overset{\rightharpoonup }{p^{\prime }}$ is the offset vertex. ${\overset{\rightharpoonup }{n}}_{pi}$ is the normalized direction vector of the element and *t* is the thickness (Fig. 6a). Due to the curve shape of the cast surface with concave shapes in some regions, a relatively great thickness may result in wrapping element shapes on the cross section, where sides of two or more elements intersect. A small thickness (e.g. 1 mm) is suggested in each step of offsetting to avoid geometric error. Offsetting elements with a small thickness is able to technically smooth the surface and further reduce the occurrence of wrapping elements [26, 27]. The accumulation of thicknesses generates the resulting thickness as required by a user.

The offsetting process generates two parallel cast surfaces without connections on opening ends. In order to create an enclosed cast model, i.e. solid-like model, nodes on opening ends on both parallel surfaces should be linked together. An edge line is created by connecting an edge node and corresponding offsetting node. Those new lines plus two opening edges and corresponding offsetting edges create two surfaces locating at each end of the cast. An enclosed cast model consists of original and offsetting surfaces plus two end surfaces. The solid cast model is developed from the enclosed model and transformed into STL format for 3D printing (Fig. 6b). This design technique develops the main structure of a cast. We use standard mechanical parts such as Velcro straps to fasten the cast when applying a cast on a patient.

### Engineering analysis

**Fig. 7:**
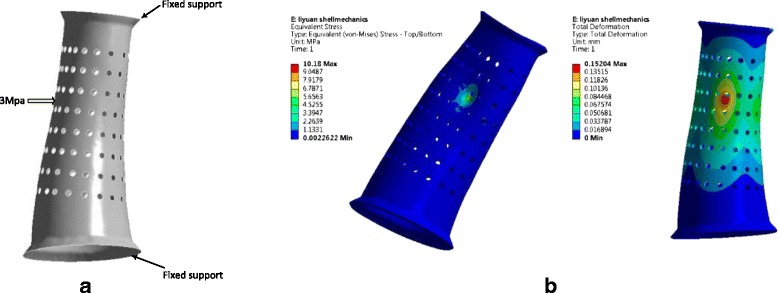
**a** The boundary condition of FEA analysis. A 3Mpa pressure is applied on the cast, and two opening edges are applied fixed support. **b** Results of finite element analysis. The maximal stress is around 10.18Mpa (left) and the maximal deformation is 0.15 mm (right). High stresses and deformities occur in the areas where mechanical loads are applied

Finite element analysis (FEA) is used to assess the engineering strength of the proposed design. The cast model is imported into ANSYS (ver. 15.0) to perform FEA. High Density Polyethylene (HDPE) or Polypropylene (PP) is utilized for 3D printing manufacturing of the cast. Mechanical properties of the material are: Young’s Modulus (E) 1,300Mpa and Poisson Ratio (v) 0.42 [28]. Basically, there are no intensive mechanical loads applied on an orthopedic cast. The accidental mechanical impact may happen when a patient hits other objects. The external load representing the unusual load is set as a 3Mpa pressure applied on areas near ventilated holes. The opening edges are fastened by Velcro straps when applying a cast on a patient. Therefore, those opening edges are set as fixed boundaries in this case (Fig. 7a). The opening gap will be bonded together when applying a cast on a patient to create a fit contact. Therefore, the FEA model set the close structure of the cast.

## Results and discussions

An intelligent designing system that integrates all above algorithms is developed by using Visualization Toolkit (VTK Kitware). Figure 6b shows a cast model for an arm orthosis. The sequence of steps of designing are outlined as follows: creating a raw cast, computing centerline, building fine cast, modeling flare, developing ventilation pattern, designing opening gap, creating the solid model. The proposed modeling algorithms are developed as an intelligent tool to assist orthopedist with few specific technical training. It takes about 20 min to perform a cast design for an orthopedic technician with few computer designing skills. In comparison, an experienced CAD (computer-aided design) designer spends more than 2.5 h to design a 3D-printed orthosis frame using a commercial software [3]. There is rare literature exploring designing orthopedic cast fabricated by 3D printing technologies. The rapid modeling technique developed in this study addresses on image-based designing technique to create a patient-specific cast. The specific techniques, such as computational geometry reference and surface pattern creation, facilitate the design process based on patients’ image data. Commercial CAD software are not user-friendly in those designs due to the complexity of geometry. Rich experiences in CAD skills and long designing time are required for an orthopaedist who uses a commercial CAD software.

A cast geometry is built from a patient’s data makes an orthosis custom-made, which creates the best fit geometry for a limb as shown in Fig. 6b. The patient-specific model would develop benefits on patient comfort and minimize the distortion after healing. A loose cast can lead the deformity of the injured limb during the healing process since the necessary correction forces are not appropriately applied. Furthermore, an ill-advisedly cast would create poor overlying skin which results in local pressure sores.

Ventilation is one of the most important concerns for a fracture patient. Ventilation pattern is designed as holed surface on the cast that improves the hygienic outcome during fracture healing and generates the lightweight structure (Fig. 6b). The specific structure avoids sweat-trapping under the cast where cutaneous complications are induced. Holed crust allows air circulation for reducing the risk of irritation and infection with higher likelihood in a damp environment where the skin is exposed. Another physical-related side benefits of the structure are lightweight and fashionable appearance. A cumbersome feature is labeled as traditional cast with a long history in the application. The novel design would improve patients’ experiences via a wearable cast with only minimal disturbance to their daily life.

**Fig. 8:**
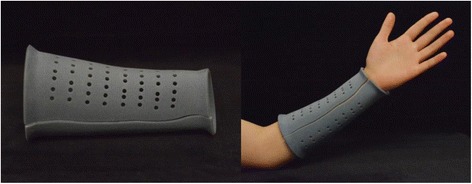
A 3D-printed cast designed by the intelligent system using rapid modelling techniques

Figure 8 displays a prototype of a 3D-printed orthosis designed by the rapid modeling technique. There is no special requirement of the type of a 3D printer for the cast fabrication. Stereolithography (SLA) printer can be used for printing the cast developed by this system. We utilize RS6000 3D printer (UnionTech, China) to print the prototype. The printing material is Polypropylene (PP) and the cast weight is about 100 g with the thickness 2 mm. The material is CFDA (China Food and Drug Administration) approved as Class I material for rehabilitation device. In comparison, a cast made from plaster has over 1 kg of the weight. The cast is also wearing friendly and can be adjusted to accommodate swelling from injured limbs by the adjustment of the opening gap. Adjustable assembly ensures dynamic clinical fit for an injured limb or limb malformation. Clinical fit is able to appropriately apply the correcting loads on a specific region and result in minimum distortion.

In spite of the lightweight and holed surface, high strength is present in the structure to resist the unusual mechanical impact. The external loads trigger high stresses concentrating on the region where forces are applied (Fig. 7b). The maximal stress approaches 11Mpa in the area of edges of a hole, where the sharp corner is more likely to develop concentrated stresses (Table 1). Despite the high stresses concentrated in some areas, the maximal stress is far lower than the yield strength of the material with around 35Mpa. The maximum displacement is less than 0.2 mm (Fig. 7b). The maximum allowable deformation can be 4.9 mm that is much greater than the simulation result. It should be noted that the deformation exceeding allowable value would result in the loose fit and failure of the correction of distortion of a limb caused by fracture [28].

**Table 1:** FEA results of the stress and deformation of a cast

Mechanical load (Mpa)	Max. stress (Mpa)	Max. deformation (mm)	Min. safety factor
3	10.2	0.15	3.44

However, the 3D scanning is a time-consuming process and a particular position of the injured limb is required for scanning in order to acquire completed data. Patients with acute fracture would be difficult to perform the required scanning. In the future study, we would design a frame to position the injured limb appropriately and facilitate scanning. In addition, the 3D printing is still a slow process as per current technologies. For example, it takes around ten hours to print the orthosis as shown in Fig. 8. Presently, fabrication of orthopedic cast using 3D printing is not an economical but affordable approach in compared to traditional methods.

## Conclusions

This study develops a rapid designing technique to create a patient-specific orthopedic cast fabricated by 3D printing technology. The newly developed cast has ventilated feature that reduce the risk of cutaneous complications occurring in traditional alternatives. In addition to ventilation feature, significantly lightweight structure is present in the cast physical model. Engineering analysis using FEA technique validate the high strength of the structure. It is a wearing-friendly and adjustable cast to create the best fit for a patient’s injured limb during the entire treatment process. The design time is short and few technical experiences are required.
